# Mechanical Performance Enhancement of 3D-Printed Temporary Dental Resin by Niobium Nanoparticle Incorporation: An In Vitro Comparative Study with Conventional Composite and 3D Permanent Materials

**DOI:** 10.3390/polym17172400

**Published:** 2025-09-03

**Authors:** Marilia Mattar de Amoêdo Campos Velo, Letícia Vendrametto Forcin, Beatriz Medola Marun, Tatiana Rita De Lima Nascimento, Mariana Souza Rodrigues, Abdulaziz Alhotan, Saleh Alhijji, Nair Cristina Brondino, Juliana Fraga Soares Bombonatti

**Affiliations:** 1 Department of Restorative Dentistry, Araraquara School of Dentistry, Sao Paulo State University (FOAr-UNESP), Humaitá, 1680, Araraquara 14801-903, SP, Brazil; 2 Department of Restorative Dentistry, Bauru School of Dentistry, University of São Paulo (FOB/USP), Alameda Dr. Octávio Pinheiro Brisolla, 9-75, Bauru 17012-901, SP, Brazil; 3 Leibniz Institute for Solid State and Materials Research, 01069 Dresden, Germany; 4 Department of Dental Health, College of Applied Medical Sciences, King Saud University, P.O. Box 10219, Riyadh 12372, Saudi Arabia; 5 Department of Mathematics, Faculty of Sciences of Bauru, São Paulo State University (UNESP), Avenida Eng. Luiz Edmundo Carrijo Coube, 14-01, Bauru 17033-360, SP, Brazil

**Keywords:** temporary resin, 3D printing, niobium, nanoparticles

## Abstract

**Background**: Three-dimensional (3D) printing has emerged as a valuable tool in dentistry for producing provisional restorations with high precision and reduced costs. However, the limited mechanical strength of temporary 3D-printed resins remains a clinical concern. This in vitro study aimed to enhance the mechanical properties of a 3D-printed temporary resin by incorporating functionalized niobium (Nb) nanoparticles and to compare its performance with a conventional resin composite and a permanent 3D-printed resin. **Methods**: Six groups were evaluated: bisacrylic resin (Protemp), resin composite (Z350), temporary 3D resin (Temp 3D), permanent 3D resin (Perm 3D), Temp 3D + 0.05% Nb, and Temp 3D + 0.1% Nb. Niobium oxyhydroxide nanoparticles were synthesized using a hydrothermal method, silanized, and incorporated into the Temp 3D at 0.05% and 0.1% by weight. The tested variables included flexural strength (FS), elastic modulus (EM), surface hardness (SH), and color stability (ΔE). **Results**: The Z350 resin showed the best mechanical results. The addition of 0.1% Nb nanoparticles significantly improved the FS, EM, and SH of the Temp 3D, reaching values comparable to the Perm 3D (*p* > 0.05). Color stability remained unaffected across all groups. **Conclusions**: These findings suggest that Nb reinforcement at a low concentration is a promising strategy for improving the performance of 3D-printed temporary restorations.

## 1. Introduction

Although modern dentistry emphasizes minimal intervention and health promotion, the loss or partial destruction of dental structures remains highly prevalent worldwide, significantly impacting individuals’ quality of life [1,2]. In this context, there is a growing demand for innovative solutions that apply conservative dentistry principles using advanced technologies. Digital tools have gained prominence by enabling more precise treatment planning and minimally invasive procedures that preserve healthy tooth structure. They also help reduce clinical time and enhance patient comfort.

While 3D printing is well established in dentistry, only a limited number of resins have been approved for permanent restorations, and these materials still exhibit insufficient resistance to masticatory forces. Temporary 3D-printed resins, on the other hand, face even greater challenges related to mechanical performance, often showing significantly inferior properties compared to resin composites commonly used for permanent restorations. This limitation directly affects their clinical reliability [3,4,5,6].

Temporary restorations play a vital role in protecting prepared teeth and ensuring patient comfort during the restorative process. They contribute to predictable and successful definitive outcomes by maintaining soft tissue health, protecting the pulp, and establishing an appropriate emergence profile, which are all prerequisites for predictable and successful definitive outcomes [7]. Compared to milled alternatives, 3D-printed provisional restorations also provide advantages such as better marginal and internal fit, improved patient acceptance, and reduced costs.

However, their limited mechanical strength and higher susceptibility to microleakage can compromise clinical performance, particularly in long-term provisional treatments and complex rehabilitations requiring extended use. In situations involving extensive provisional restorations or single-unit cases, these materials must withstand masticatory forces without fracturing or dislodging [4,5,6,8]. Therefore, enhancing the performance of provisional dental resins is essential, particularly to ensure reliable short- to medium-term clinical durability in cases where temporary restorations must withstand functional demands for extended periods.

Nanotechnology has emerged as a promising strategy to enhance the properties of resin-based dental materials by incorporating fillers, such as fibers and nanoparticles, thereby improving their mechanical and physical properties [9,10]. However, optimizing printable resin performance requires careful selection of filler type, size, and concentration, as these directly affect both printability and the material’s final behavior.

Among potential nanomaterials, niobium (Nb) nanoparticles have garnered attention due to their exceptional mechanical strength, corrosion resistance, and proven biocompatibility. Incorporating Nb nanoparticles into resin composites has been shown to improve key mechanical properties, such as flexural strength, elastic modulus, and surface hardness [11], thereby enhancing structural integrity and long-term performance without compromising handling or aesthetic features. In addition, the use of niobium oxide as a filler is particularly attractive from a practical and economic perspective, since Brazil holds the world’s largest niobium reserves, ensuring wide availability and potential cost reduction [11,12]. Thus, beyond its favourable biological and mechanical profile, niobium represents a sustainable and accessible alternative for developing advanced dental materials.

However, metal oxides typically exhibit low surface reactivity, which can lead to weak bonding with the resin matrix due to unstable chemical interactions and limited mechanical interlocking. To overcome this, surface functionalization of oxide particles improves chemical stability and promotes better dispersion within the composite, reducing agglomeration [13]. In this regard, functionalized niobium oxyhydroxide is particularly promising, as it provides numerous active sites for chemical interaction, potentially enhancing composite performance [11].

Although the incorporation of Nb nanoparticles into resin materials has shown encouraging results [9,10], there are still gaps regarding the mechanisms and optimal conditions for their use in 3D-printed temporary resins. Therefore, this study aimed to reinforce a 3D-printed temporary resin with functionalized Nb nanoparticles to increase its durability and to compare the mechanical performance of the reinforced temporary resin with that of a conventional resin composite and a permanent 3D-printed resin.

## 2. Materials and Methods

### 2.1. Experimental Design

This in vitro study evaluated two factors: (1) restorative material, including Temp 3D (Temporary CB Resin, FormLabs, Somerville, MA, USA), bisacrylic resin (BIS; Protemp™, Solventum, Saint Paul, MN, USA), microhybrid resin composite (Z350; Filtek Z350, Solventum), and 3D printing resin for definitive restorations (Perm 3D; Permanent Crown Resin, FormLabs); and (2) the following response variables: flexural strength (FS), elastic modulus (E), surface hardness (SH), and colour stability (ΔE).

### 2.2. Synthesis of Niobium Nanoparticles and Development of 3D Printing Resins

The synthesis of niobium oxyhydroxide nanoparticles was conducted as previously described by Obeid et al. [11]. Briefly, niobium ammonium oxalate precursor was reacted with sodium hydroxide under controlled pH and temperature conditions. The resulting mixture underwent ageing and washing steps to yield a precipitate, which was then dried and sieved. The resulting nanoparticles were salinized using (3-mercaptopropyl) trimethoxy silane (MPTMS) via a reflux reaction in xylene, with mechanical stirring under a nitrogen atmosphere. Following washing and drying, the salinized nanoparticles were characterized through morphological and compositional analyses, including assessment of salinization and dispersion using Fourier-transform infrared spectroscopy (FTIR) and field-emission scanning electron microscopy (FEGSEM) coupled with energy-dispersive X-ray spectroscopy (EDS). The niobium synthesis and functionalization protocol used in this study was based on our prior work [11] to ensure consistency and reproducibility. This study represents the first application of these nanoparticles to 3D-printed temporary dental resins, focusing on their potential to enhance mechanical properties and address clinical needs for improved durability. In the present study, these previously synthesized and functionalized niobium nanoparticles were incorporated into the 3D printing resins.

### 2.3. Incorporation of Nb Nanoparticles and Specimens’ Preparation

Nb nanoparticles were weighed to achieve concentrations of 0.05% and 0.1 wt% corresponding to the mass of the respective resin composite specimens. Then, nanoparticles were incorporated into the resin using an ultrasonic probe sonicator (Eco-Sonics QR850, 850 W, 20 kHz, acoustic chamber).

**Figure 1:**
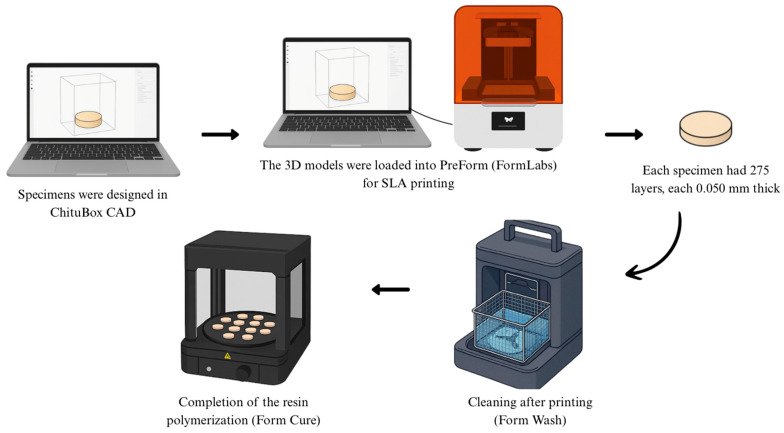
Diagram of the printing procedure.

All specimens fabricated with the 3D printing resins, Temp 3D or Perm 3D, were initially designed using CAD software (ChituBox Basic V1.9.5) and subsequently imported into PreForm software for stereolithography (SLA) 3D printing. Printing was performed on a Form3B+ printer (FormLabs) following the manufacturer’s recommended parameters and guidelines. For all specimens, the print orientation was set at 90°, with a total print time of 1 h and 28 min. Each specimen was composed of 275 layers, with a layer thickness of 0.050 mm (Figure 1).

In this study, automated printing settings were employed to ensure reproducibility and minimize variability. This approach allowed for a clear assessment of the effects of niobium nanoparticle incorporation on the mechanical properties of the resin without confounding variables. By focusing on a standardized methodology, we aimed to isolate the impact of nanoparticle reinforcement on the material’s performance.

### 2.4. Sample Size Calculation

The sample size was calculated based on FS using the GLIMMPSE software 3.0.0 (http://glimmpse.samplesizeshop.org/, accessed on July 2024). A general linear model with one between-subjects factor (treatment) was applied. The Hotelling–Lawley trace statistic was used to adjust the degrees of freedom. Statistical power was set at 80%, with a significance level of α = 0.05. Because there are still limited studies directly comparing modified 3D-printed resins with conventional restorative and provisional materials, the group means used for the calculation were based on previous study that modified 3D-printed resin [3]. In that study, the following mean FS values (MPa) were obtained: Z350 (105.1), BIS (27.9), and Temp 3D (67.15), while the manufacturer’s reported value of 116 MPa was used for the permanent 3D resin. Variance was modelled with an assumed SD = 20 MPa, consistent with reported variability for resin-based materials [14]. To ensure robustness, the simulations also allowed the means to vary from half to twice the estimated values and the variance up to twice the assumed SD. Based on these parameters, a total sample size of 54 specimens (*n* = 9 per group) was determined.

### 2.5. Flexural Strength (FS) and Modulus of Elasticity (E) Procedure

Twelve composite bar-shaped samples measuring 8 × 2 × 2 mm^3^ (*n* = 12) were fabricated for each group, in accordance with ISO 4049, with an adjustment in specimen length to prevent overexposure or insufficiently cured regions, considering the diameter of the LED curing unit [15,16].

For the composite and bisacrylic resins, the specimens were prepared using a two-part Teflon mold. Specimens were first designed using CAD software (ChituBox Basic V1.9.5) and then transferred to PreForm software. These were printed via SLA using a Form3B+ printer (FormLabs), following the manufacturer’s recommended parameters and guidelines.

All samples were stored in dark containers at a controlled temperature of 37 °C for 24 h prior to testing. The three-point bending test was conducted using a universal testing machine (Instron 5943, Norwood, MA, USA) equipped with a 500 N load cell, operating at a constant crosshead speed of 0.5 mm/min. Specimen dimensions were measured using a digital calliper prior to testing and entered the testing software. Flexural strength values were calculated using the following equation:

$$FS=\frac{3FI}{2bd^{2}}$$

where FS is the fracture load, F is the loading force at the fracture point, I is the span length between the supports (6 mm), b is the specimen width (mm), and d is specimen thickness (mm).

To calculate the modulus of elasticity, the following formula was used:

å= I3 × F1 /4fbh3

where å is the modulus of elasticity, *I* the span length between the supports (6 mm), b (2 mm) is the width of the specimen and h (2 mm) is the height of the specimen, *F*1 (N) the is the applied load within the elastic region and f (mm) is the deflection of the bar in the elastic phase [15,16].

### 2.6. Knoop Surface Hardness (SH) Procedure

To measure the surface hardness (SH) of the groups, flat disc specimens measuring 10 mm in diameter and 2 mm in thickness were prepared for each group (*n* = 12). For the bis-acrylic and resin composite groups, discs were fabricated using two-part Teflon molds, positioned between two glass slides with polyester strips in between. Resin composite specimens were light-cured for 20 s using a polywave LED light-curing unit (395–480 nm; Valo Grand, Ultradent Products, Inc. South Jordan, UT, USA). with an intensity of 1000 mW/cm^2^.

Specimens made from the 3D printing resins Temp 3D or Perm 3D were digitally designed with the same dimensions using CAD software (ChituBox Basic V1.9.5), transferred to PreForm software, and printed via SLA using a Form3B+ printer (FormLabs), following the manufacturer’s recommended parameters.

All specimens were stored in dark containers at a controlled temperature of 37 °C for 24 h prior to testing. After this period, the specimens were mounted on epoxy resin discs using utility wax and polished using a sequence of abrasive papers (grit sizes 600 and 1200), followed by polishing with felt discs and 0.5 µm diamond paste (Buehler Ltd., Lake Bluff, IL, USA).

Surface hardness was measured by making three indentations at the center of each specimen, spaced 100 µm apart, using a microhardness tester with a Knoop diamond indenter (50 g load for 15 s; Buehler Ltd.). Surface hardness values were expressed in Knoop Hardness Number (KHN), calculated based on the measured dimensions of the indentations on each specimen.

### 2.7. Colour Stability (ΔE) Procedure

For the color analysis, the specimens identical in shape to those used for the surface hardness test—flat discs measuring 10 mm in diameter and 2 mm in thickness (*n* = 10)—were prepared for each group. For the bis-acrylic and resin composite groups, discs were fabricated using two-part Teflon molds, positioned between two sliding glass plates with polyester strips in between. Resin composite discs were light-cured for 20 s using a polywave LED curing unit (wavelength range: 395–480 nm; Valo Grand, Ultradent Products, Inc., South Jordan, UT, USA) at an intensity of 1000 mW/cm^2^.

Specimens for the 3D printing resin groups were printed with the same dimensions using Temp 3D on a Form3B+ SLA 3D printer, following the manufacturer’s instructions.

All specimens were stored in dark containers at a controlled temperature of 37 °C for 24 h prior to testing. After this period, the specimens were mounted on epoxy resin discs using utility wax and polished with a sequence of abrasive papers (600 and 1200 grit), followed by a felt disc and 0.5 µm diamond polishing paste (Buehler Ltd., Lake Bluff, IL, USA).

Before taking colour measurements, the spectrophotometer was calibrated according to the manufacturer’s instructions. Color evaluation was performed at two time points using the CIEL*a*b* system:

- (P0) after 24 h of specimen polymerization, and
- (P1) after eight days of immersion in distilled water at 37 °C.

Three measurements were taken for each specimen, following the methodology described by Scotti et al. (2020) [3].

ΔE was calculated using the following equation:

$$\Delta E=\sqrt{{\left({\Delta L}^{*}\right)}^{2}+({\Delta a}^{*})+{\left({\Delta b}^{*}\right)}^{2}}$$

where

- ΔL*, Δa* and Δb* represent the color differences observed between P0 and P1.

### 2.8. Statistical Analysis

Statistical analyses were performed using R software 4.3.2 (R Core Team, 2024), with a significant level of α = 0.05. To evaluate the effect of group on flexural strength and elastic modulus, linear models were fitted to the data. For surface hardness, a generalized Linear model (GLM) with a Gamma distribution and an inverse link function was applied.

For all models, the assumption of homoscedasticity (constant variance) was assessed using plots of residuals versus fitted values plots. Residual normality was evaluated Q–Q plots with simulated envelopes and the Shapiro–Wilk test (for linear models). For the linear models, group differences were tested using analysis of variance (ANOVA), while deviance analysis (ANDEV) was used for the GLM. Pairwise comparisons were adjusted using Tukey’s method.

To assess the effect of group on ΔE, a generalized linear model with a Gamma distribution and a log link function was employed. As with the other models, residual normality was examined using Q–Q plots with simulated envelopes, and homoscedasticity was assessed via by residuals’ versus fitted values plots. Deviance analysis was used to test the significance of effects. All statistical procedures were conducted using R software 4.3.2 (R Core Team, 2024), with adopting a significant level of α = 0.05.

## 3. Results

The composition of the resin composites is summarized in Table 1. These compositional features describe the materials under investigation and are relevant for the interpretation of the results presented herein.

### 3.1. Flexural Strength and Modulus of Elasticity

To analyze the effect of groups on the FS, a general linear model was fitted to the data. The assumption of residual normality was evaluated using the Shapiro–Wilk test and Q–Q plots with simulated envelopes, while homoscedasticity (constant variance) was assessed through plots of residuals versus fitted values. ANOVA revealed a statistically significant difference among groups (*p* < 0.0001), leading to the rejection of the null hypothesis of equal means. The effect size was η^2^ = 0.84 (95% bootstrap CI [0.76, 0.88]), indicating a large effect size according to Cohen’s classification. Following this, pairwise comparisons were conducted using Tukey’s method with *p*-values adjusted accordingly.

**Figure 2:**
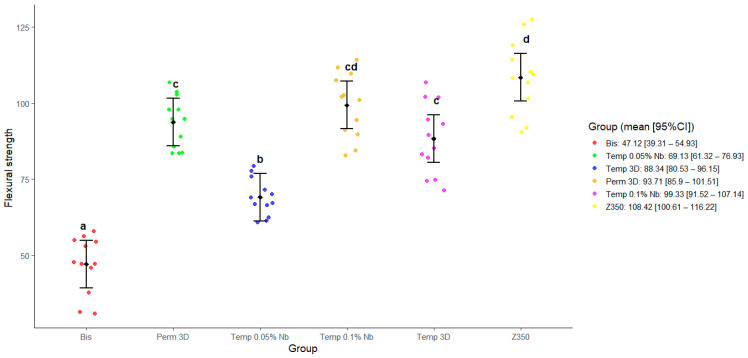
Estimated marginal means for the flexural test as predicted by the model, with 95% confidence intervals. Different letters indicate statistically significant differences (*p* < 0.05).

The tests showed no evidence to reject the null hypothesis of equal FS for the following group comparisons: Perm 3D and Temp 0.1% Nb (*p* = 0.74), Perm 3D and Temp 3D (*p* = 0.77), Temp 0.1% Nb and Z350, and Temp 0.1% Nb and Temp 3D (*p* = 0.09). These results indicate no significant differences in FS between these groups, suggesting similar performance at the α = 0.05 level. All other pairwise comparisons yielded *p*-values below 0.0002, indicating statistically significant differences. These results are summarized in the graph shown in Figure 2.

**Figure 3:**
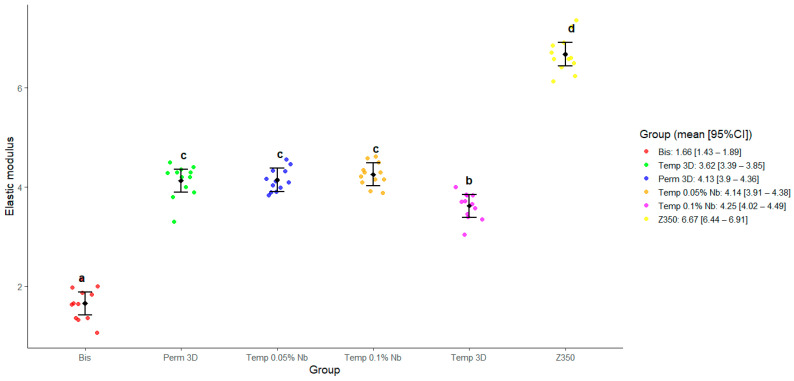
Estimated marginal means and 95% confidence intervals for the elastic modulus as predicted by the model. Different letters indicate statistically significant differences (*p* < 0.05).

For modulus of elasticity (E), ANOVA also indicated the rejection of the null hypothesis of equality among the groups (*p* < 0.0001), suggesting that group membership influenced the modulus of elasticity. The effect size was η^2^ = 0.96 (95% bootstrap CI [0.95, 0.98]), indicating a large effect size according to Cohen’s classification. Subsequent pairwise comparisons were made using Tukey’s test. The results of these tests showed no evidence to reject the null hypothesis of equal elasticity moduli for the comparisons between Perm 3D and Temp 0.05% Nb (*p* = 0.99), Perm 3D and Temp 0.1% Nb (*p* = 0.9), and Temp 0.05% Nb and Temp 0.1% Nb (*p* = 0.94). The null hypothesis of equality between the expected means was rejected for all other pairwise comparisons (*p*-value less than 0.0011 for all). The results of the pairwise comparisons are summarized in the graph presented in Figure 3.

**Figure 4:**
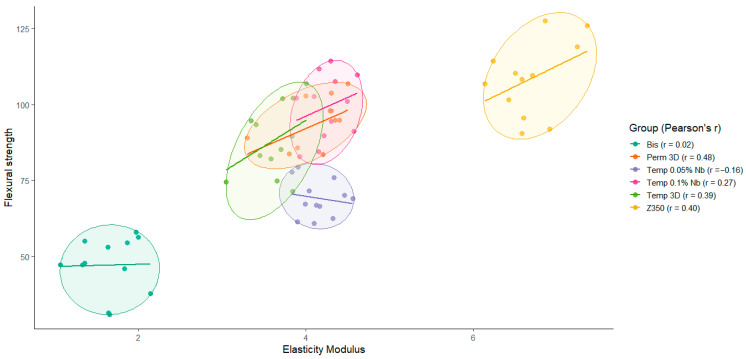
Combined analysis of flexural strength and elastic modulus. The figure illustrates the association between elastic modulus and flexural strength, highlighting the relationship observed between the Temp 3D with 0.01% Nb group and the Perm 3D group.

Figure 4 presents the joint distribution of flexural strength and elastic modulus values for the six experimental groups. While no important correlation is observed between FS and E in the Bis (r=0.02) and Temp 0.05% Nb (r=−0.16) groups, a positive correlation is evident in the remaining groups, especially for groups Perm 3D (r=0.48), Temp 3D (r=0.39) and Z350 (r=0.40). This suggests that, in these groups, higher flexural strength values are generally associated with higher elastic modulus values.

### 3.2. Surface Hardness

**Figure 5:**
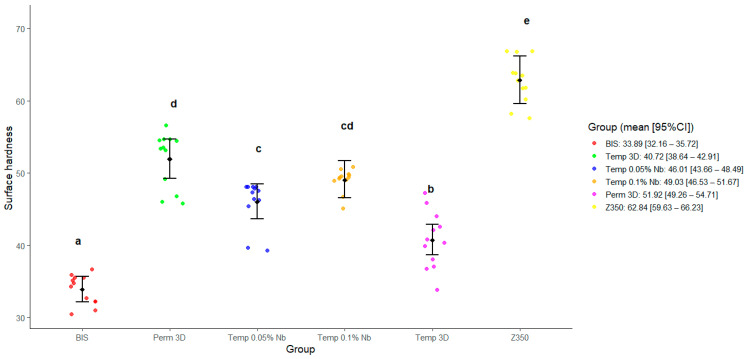
Estimated marginal means and 95% confidence intervals for the surface hardness as predicted by the model. Different letters indicate statistically significant differences (*p* < 0.05).

Figure 5 illustrates the distribution of surface hardness values across the experimental groups. The shape of the density contours indicates asymmetries in the distributions for the Bis, Temp 0.1% Nb, Temp 0.05% Nb, and Perm 3D groups. These groups also showed concentrations of values above the mean. Despite the distributional differences, the variances among groups appear relatively homogeneous.

The deviance analysis showed a statistically significant group effect (*p* < 0.0001). The effect size was η^2^ = 0.07 (95% bootstrap CI [0.01, 0.17]), indicating a medium effect size according to Cohen’s classification. The Bis and Temp 3D groups exhibited the lowest mean hardness values. The Temp 0.1% Nb group displayed the lowest variance, while the highest variances were observed in the Perm 3D and Temp 3D groups.

Statistical testing revealed no evidence to reject the null hypothesis of equal hardness values between Perm 3D and Temp 0.1% Nb (*p* = 0.31), and between Temp 0.05% Nb and Temp 0.1% Nb (*p* = 0.20). For all other pairwise comparisons, the null hypothesis was rejected with *p*-values below 0.0005 in each case, indicating statistically significant differences in surface hardness among those groups.

### 3.3. Colour Stability

**Figure 6:**
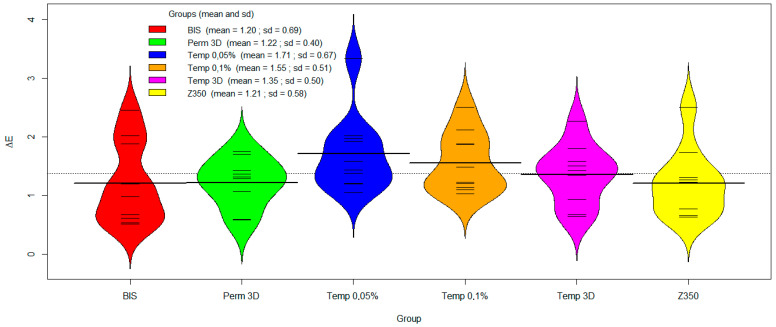
Beanplot for colour stability.

The Beanplot presented in Figure 6 illustrates the distribution of ∆E values across experimental groups. Ticker lines represent individual observations and group means are highlighted with bold lines. Except the Temp 3D resin, all distributions exhibit noticeable asymmetry. While the Perm 3D group shows a concentration of ∆E values above the mean, the other four groups display concentrations below the mean. The plot also suggests variances among the groups. The Bis and Temp 0.05% Nb groups exhibited the lowest mean ∆E values, with the smallest variance also found in the Temp 0.05% Nb group. The results of the ANDEV indicated that the null hypothesis of equality among the groups was not rejected (*p* = 0.27). In other words, the data suggests that the groups behaved similarly regarding ∆E. The effect size was η^2^ = 0.17 (95% bootstrap CI [0.05, 0.36]), indicating a large effect size according to Cohen’s classification.

## 4. Discussion

Three-dimensional (3D) printing is a rapidly evolving technology that has been widely adopted in modern dentistry. However, technical challenges remain—such as high polymerization shrinkage, low degree of conversion, and limited mechanical strength—which constrain the clinical applicability of 3D printing resins [7]. In response, manufacturers are continually working to enhance these materials for definitive intraoral use, as exemplified by the Permanent Crown Resin from FormLabs used in this study.

SLA printing is currently one of the most recommended technologies for dental applications due to its high precision and seamless integration with digital workflows, including intraoral scanning, computed tomography, and CAD software [8]. Despite progress in developing permanent 3D printing resins, the scientific literature still lacks robust evidence to fully support these recommendations.

In this study, the conventional resin Z350 exhibited significantly superior FS values (*p* = 0.09) and across all evaluated variables. The fragility often observed in SLA-printed structures can be attributed to the heterogeneous polymer network, which results from the uneven diffusion of unreacted monomers during vitrification and rapid reaction kinetics [10]. This phenomenon likely explains the inferior mechanical performance of printed resins compared to Z350.

In this context, it is important to highlight that printing parameters such as layer thickness, exposure time, print orientation, and post-curing conditions may influence the final properties of additively manufactured materials [14]. In the present study, however, these parameters were kept constant and standardized through the printer’s automated settings. This methodological choice was intended to minimize variability, ensure reproducibility, and enable controlled comparisons across the experimental groups, thereby allowing the influence of nanoparticle incorporation to be more clearly assessed.

Results for FS, E, and SH showed that the bisacrylic resin Protemp exhibited the lowest values, while Z350 demonstrated the highest, corroborating previous findings from our research group [3]. Reinforcing of 3D printing resins with nanoparticles, has emerged as a promising strategy to improve mechanical performance, enhance durability, and broaden clinical application [17]. Due to their nanoscale dimensions and high surface area, niobium nanoparticles enhance interaction with the polymer matrix, improving mechanical strength [18]. According to Obeid et al., 2024 [11], FTIR spectra of the functionalized particles exhibited the same profile as the non-functionalized material, but with additional bands confirming silane incorporation. A distinct peak at 2953 cm^−1^ was associated with C–H stretching of the organic moiety, while absorptions at 1124 cm^−1^ and 1012 cm^−1^ indicated Si–C and Si–O bonds, respectively, supporting the successful attachment of MPTMS to the niobium matrix. SEM micrographs showed clustered yet relatively homogeneous structures, and the presence of silicon detected in the EDS analysis further corroborated the functionalization process [11]. In this study, low concentrations (0.05% and 0.1%) were chosen to promote optimal particle dispersion and prevent agglomeration, which can negatively affect material properties [19].

The addition of 0.1% functionalized Nb nanoparticles to the Temp 3D yielded FS values comparable to those of the Z350 resin, demonstrating significant mechanical reinforcement. The incorporation of 0.1% niobium nanoparticles into the temporary 3D resin resulted in mechanical properties comparable to those of permanent 3D resins. This improvement has significant clinical implications, particularly for extended provisional treatments in complex rehabilitations, where enhanced durability can reduce restoration failures, minimize patient discomfort, and decrease treatment interruptions. These findings highlight the potential of niobium nanoparticle reinforcement to address critical clinical challenges in temporary restorations. This finding is in accordance with reports in the literature suggesting that moderate nanoparticle concentrations are more effective than higher loadings, which can lead to agglomeration and create weak zones within the polymer matrix [19]. A pilot study conducted as part of this project found that higher niobium concentrations (0.5%, 1%, and 2.5%) were associated with decreased mechanical performance.

Data from Figure 2 and Figure 5 show that the Temp 3D, with 0.1% niobium nanoparticles, achieved mechanical properties like the permanent resin, including a notable increase in elastic modulus in both niobium-reinforced groups. Correlation analysis between FS and E revealed no significant correlation in the Bis and Temp 0.05% Nb groups. In contrast, the other groups showed a positive correlation, indicating that higher FS values are generally associated with greater stiffness.

FS is a fundamental mechanical property of the materials used in oral rehabilitation, since masticatory fatigue can lead to fractures in temporary dental restorations, causing discomfort to the patient, in addition to increasing costs and prolonging treatment time. A higher flexion capacity is essential for the clinical performance of temporary prostheses [6]. Thus, the use of temporary crowns with greater resistance to flexion can ensure greater durability in the face of masticatory forces, reducing the risk of fractures and ensuring a rehabilitation period without aesthetic, inflammatory, and painful complications for the patient. In this context, reinforcement with Nb nanoparticles becomes interesting, due to its ability to increase the flexural strength, elastic modulus, and surface hardness of the material [10,11].

At the microstructural level, functionalized niobium acts as a physical reinforcement agent by filling voids within the polymer matrix, enhancing internal cohesion, and promoting uniform stress distribution. This mechanism improved energy dissipation under masticatory forces, reducing the risk of crack propagation and fracture [9]. Additionally, niobium’s photocatalytic properties may enhance polymerization kinetics, potentially reducing curing time and improving the final physic-mechanical properties [20].

Provisional restorations are primarily intended for short-term use during the fabrication of definitive restorations. However, in complex clinical scenarios, such as extensive prostheses, multi-unit cases, or patients with altered occlusion, these temporary restorations must demonstrate sufficient mechanical strength to withstand functional loads. In addition to their protective role, they serve as important diagnostic tools, allowing clinicians to assess both functional performance and aesthetic outcomes before final restoration placement. Ensuring adequate mechanical resistance is therefore essential to prevent failures, protect underlying structures, and maintain patient comfort during the provisional phase.

Catastrophic failures have been reported to occur more frequently in 3D-printed provisional restorations compared to conventional ones [21], emphasizing the need for long-term in vivo studies to determine whether improvements in mechanical properties translate to meaningful clinical benefits.

In our study, FS values for provisional resins have been reported in the range of approximately 50–80 MPa. All groups exceeded the minimal acceptable threshold for provisional fixed dental prosthesis (FDP) materials, defined as 50 MPa according to ADA-ANSI Specification #27 [22,23]. The addition of 0.1% Nb resulted in FS values approaching those of permanent resins, suggesting that the reinforced temporary material may better resist functional loads over time; however, the clinical durability of this reinforcement still requires confirmation through long-term in vivo studies.

On the other hand, colour stability continues to represent a limitation of 3D printing resins, being influenced by factors such as chemical composition, photoinitiator type, pigment quality, and exposure to oral environmental conditions [3,24]. The inherent permeability of the polymer facilitates the uptake of staining agents, thereby contributing to material discolouration. In the present study, no significant differences in ΔE values were observed among the evaluated groups, including those modified with niobium nanoparticles. Considering the clinically acceptable threshold of ΔE = 6.8 and the perceptibility threshold of ΔE = 3.7 [25], all groups exhibited values below these limits, indicating that the materials maintained colour within clinically acceptable aesthetic standards. Chemically inert and resistant to oxidation, Nb nanoparticles exhibit minimal reactivity with oral environmental agents such as acids and dietary pigments [19]. Furthermore, due to their nanometric size, they allow high light transmittance and uniform dispersion in the resin matrix without compromising aesthetic [9,24].

Overall, improving the mechanical properties of provisional restorations is essential to enhance their clinical longevity, thereby reducing the incidence of fractures, the need for replacements, and chairside interventions. Such improvements represent a significant long-term benefit for both patients and clinicians. From an economic view, the incorporation of niobium oxide as a filler is particularly advantageous. Brazil possesses the largest global reserves of Nb, which ensures wide availability and a relatively low cost compared with other metallic or ceramic nanoparticles commonly employed in dental materials. Consequently, the additional cost associated with niobium nanoparticle reinforcement is expected to be minimal, especially when balanced against the potential reduction in restoration failures and the consequent need for retreatments [26,27]. In this context, the use of Nb emerges as a practical and economically sustainable strategy to enhance the performance of provisional resins while preserving feasibility for clinical application.

In summary, the incorporation of 0.1% Nb nanoparticles into Temp 3D appears to be a promising strategy for enhancing physical and mechanical properties without adversely affecting optical characteristics. Nevertheless, this study is subject to limitations inherent to in vitro investigations, as it does not account for clinically relevant conditions such as the presence of saliva, fluctuations in pH, and biofilm formation—factors that can significantly influence material performance [28]. These considerations underscore the necessity for complementary in vivo studies [28,29]. However, further studies are needed to explore the potential of this approach for definitive restorative restorations and to support the development of more durable, high-performance 3D-printed resin composites.

## 5. Conclusions

- The evaluated 3D-printed resins exhibited lower mechanical strength than the conventional resin composite Z350 but outperformed the bisacrylic resin.
- The 3D-printed resin designed for permanent restorations demonstrated superior mechanical performance compared to the temporary 3D resin.
- The incorporation of 0.1% functionalized niobium nanoparticles into the 3D-printed temporary resin resulted in a significant improvement in its mechanical properties. Although long-term durability was not directly assessed in this study, the findings suggest a promising potential for the development of more resistant and clinically reliable provisional restorations for extended use.
- All tested resins exhibited satisfactory color stability over time.

## Figures and Tables

**Table 1:** Composition of the resins used.

Resins	Manufactures	Composition
Temp 3D	Form Labs	Esterification products of ethoxylated 4,4’-isopropylidenediphenol and 2-methylprop-2-enoic acid (50–75%); diphenyl (2,4,6-trimethylbenzoyl)phosphine Oxide (photoinitiator) (<2.5%).
BIS (ProtempT)	Solventum	Dimethacrylate (BISEMA 6), silane-treated amorphous silica, reaction products of 1,6-diisocyanatohexane with 2-(2-methacryloxy)ethyl-6-hydroxyhexanoate, 2-hydroxyethyl methacrylate (HEMA), silane-treated silica, ethanol, 2,2’-((1-methylethylidene)bis(4,1 phenyleneoxy))biset hyl diacetate, benzyl-phenyl barbituric acid, and tert-butyl peroxy-3,5,5-trimethylhexanoate.
Z350	Solventum	Bis-GMA, Bis-EMA, UDMA, TEGDMA, Zirconia, and silica fillers. Silane and pigments. Loading percentage by weight: 82% (5–20 nm nonagglomerated silica and 5–20 nm zirconia/silica nanoagglomerate. 0.6–1.4 um agglomerated particles).
Perm 3D	Form Labs	Esterification products of ethoxylated 4,4’-isopropylidenediphenol and 2-methylprop-2-enoic acid (50–75%), and diphenyl(2,4,6-trimethylbenzoyl)phosphine oxide as a photoinitiator (<3%).

## Data Availability

The data presented in this study are available on request from the corresponding author.
